# Redox Regulation of Immunometabolism in Microglia Underpinning Diabetic Retinopathy

**DOI:** 10.3390/antiox13040423

**Published:** 2024-03-29

**Authors:** Luwei Cai, Mengxue Xia, Fang Zhang

**Affiliations:** 1 National Clinical Research Center for Eye Diseases, Shanghai General Hospital, Shanghai Jiao Tong University School of Medicine, Shanghai 200080, China; lwcai20010530@stu.suda.edu.cn (L.C.); thesnowdream@sjtu.edu.cn (M.X.); 2 Shanghai Key Laboratory of Ocular Fundus Diseases, Shanghai 200080, China; 3 Shanghai Engineering Center for Visual Science and Photomedicine, Shanghai 200080, China; 4 Shanghai Engineering Center for Precise Diagnosis and Treatment of Eye Diseases, Shanghai 200080, China

**Keywords:** redox, immunometabolism, microglia, diabetic retinopathy

## Abstract

Diabetic retinopathy (DR) is the leading cause of visual impairment and blindness among the working-age population. Microglia, resident immune cells in the retina, are recognized as crucial drivers in the DR process. Microglia activation is a tightly regulated immunometabolic process. In the early stages of DR, the M1 phenotype commonly shifts from oxidative phosphorylation to aerobic glycolysis for energy production. Emerging evidence suggests that microglia in DR not only engage specific metabolic pathways but also rearrange their oxidation-reduction (redox) system. This redox adaptation supports metabolic reprogramming and offers potential therapeutic strategies using antioxidants. Here, we provide an overview of recent insights into the involvement of reactive oxygen species and the distinct roles played by key cellular antioxidant pathways, including the NADPH oxidase 2 system, which promotes glycolysis via enhanced glucose transporter 4 translocation to the cell membrane through the AKT/mTOR pathway, as well as the involvement of the thioredoxin and nuclear factor E2-related factor 2 antioxidant systems, which maintain microglia in an anti-inflammatory state. Therefore, we highlight the potential for targeting the modulation of microglial redox metabolism to offer new concepts for DR treatment.

## 1. Introduction

Diabetic retinopathy (DR) is a common secondary microvascular and neurodegenerative complication of diabetes mellitus, occurring in approximately 30% to 40% of diabetic patients [1,2,3]. As is estimated by 2030, the number of patients influenced by DR will reach 191 million [4]. DR is widely recognized as the leading cause of diabetes-related visual impairment among the working-age population all over the world [5,6]. If not prevented and controlled effectively, it will bring a great burden to society and the economy.

Recently, the administration of intravitreal injections of anti-vascular growth factor (VEGF) agents has demonstrated efficacy in mitigating retinal neovascularization and subsiding diabetic macular edema, representing significant advancements in therapeutic intervention [7]. However, anti-VEGF therapy is only effective in a fraction of patients with DR, requiring regular intravitreal injections, and its effects may wear off after a few years [8,9]. Therefore, as the incidence of diabetes continues to rise globally, we need to develop new treatments for this disease to prevent the early progression of DR. Nevertheless, the pathogenic mechanisms of DR have not been clarified yet, which hinders the development of new, effective treatments.

DR was originally described as a prototypical microvascular disease, manifesting vascular alterations such as microaneurysms in its advanced stage [10]. However, it is increasingly recognized that neuroinflammation and dysregulation of the immune system contribute significantly to the pathogenesis of DR at an early stage [11,12,13]. Microglia, the primary mediator of the inflammatory response, are present as the effector cells of innate immunity in the retina [14]. They are highly plastic and perform a diverse range of specialist functions in DR, including phagocytosis and removing toxic aggregates that contribute to inflammation and eventually neurodegeneration in DR [15]. Therefore, exploration of the immune regulatory mechanisms of microglia in the early stage of DR is expected to offer innovative insights for the development of preventive strategies against the onset of early-stage DR.

It is now recognized that microglia can be divided into two main phenotypes [16,17]. The classical, pro-inflammatory M1 phenotype is characterized by elevated production of reactive oxygen species (ROS) and cytokines such as interleukin (IL)-1β, IL-6, IL-33, and tumor necrosis factor (TNF)-α, while the anti-inflammatory M2 phenotype mitigates neuroinflammation by secreting IL-4, IL-10, and transforming growth factor (TGF)-β, promoting debris clearance, oxidative stress resolution, and tissue remodeling [18,19,20,21]. Recently, the field of immunometabolism has attracted considerable attention in the investigation of microglial polarization regulation. Immunometabolism is defined as the intracellular metabolic pathway that determines the fate and function of immune cells [22]. It describes changes in the interactions between immune and metabolic pathways during stress, which is a complex, dynamic process involving many regulators or signals [23,24]. It has been discovered that following activation, immune cells, especially microglia, reconfigure their redox systems, leading to the reprogramming of metabolism [25,26,27]. A great number of reports have shown that oxidative stress in microglia plays a crucial role in the pathogenesis of DR [28,29]. Neuroinflammation and excessive oxidative stress work together to cause damage to retinal blood vessels and neurons [30], eventually leading to neuronal degeneration, which is an early manifestation of DR [31]. However, the specific mechanism underlying the metabolism and redox regulation of microglia in DR remains to be further elucidated.

In this review, we initially introduce the concept of microglia polarization, followed by an analysis of the metabolic regulation of microglia polarization through immunometabolism in DR. Subsequently, we provide comprehensive roles played by redox systems (including the NADPH oxidase (NOX) system, the thioredoxin (TRX) system, and the nuclear factor E2-related factor 2 (NRF2) pathway) in the immunometabolism process associated with DR. Finally, we summarize the impact of redox signals on the metabolic status of microglia, along with potential therapeutic targets based on the redox regulation of immunometabolism, with the intention of offering innovative therapeutic insights and guidance for the clinical management of DR.

## 2. Immunometabolism in Microglia Polarization in DR

### 2.1. Phenotype of Microglia

Microglia can respond to endogenous stimuli after infection or injury and show pathogenic or protective effects. Conventionally, microglia can be divided into two distinct phenotypes: classically activated microglia, also known as the pro-inflammatory phenotype (M1 type), and alternately activated microglia, also known as the anti-inflammatory phenotype (M2 type). Different phenotypes of microglia exhibit completely distinct morphology, surface markers, secretory products, and functions.

The polarization of microglia towards the M1 phenotype is known as the classical activation pathway. Damage-induced cell debris, lipopolysaccharides (LPSs) derived from bacteria, and pro-inflammatory cytokines such as IL-1β and TNF-α can activate microglia and promote their polarization toward the M1 phenotype [32,33,34]. At this point, they begin to express unique surface markers such as cluster of differentiation (CD)14, CD16, CD32, CD80, and CD86. Upon activation, M1 microglia take on amoeboid morphology and acquire high phagocytosis and motility for killing foreign pathogens [17,35]. In the meantime, activated microglia, like peripheral macrophages, release various inflammatory factors (TNF-α, IL-1β, IL-6, IL-12, and IL-23), chemokines, redox molecules (ROS, phagocytosis oxidase, inducible nitric oxide synthase (iNOS)), cell adhesion molecules (ICAM-1), VEGF, and MHC-II [36]. Under physiological circumstances, these pro-inflammatory mediators help remove toxic aggregated proteins and cellular debris from the retina. However, in the case of pathological conditions such as DR, overactivated M1-type microglia release large amounts of neurotoxic inflammatory factors (TNF-α, IL-1α, IL-1β, IL-6, NO, hydrogen peroxide, superoxide anion, and glutamate), and these pro-inflammatory factors induce leukocyte infiltration into the retinal vasculature, leading to vascular inflammation and disruption of the blood retinal barrier (BRB) [37,38]. Early BRB breakdown is a typical event in the early stage of DR, leading to increased exposure of microglia to extracellular signals. Thereby initiating a pernicious cycle of inflammation.

The polarization of microglia towards the M2 phenotype is known as the alternative activation pathway. M2 microglia are activated by anti-inflammatory cytokines such as IL-4, IL-10, and IL-13 [39,40,41]. Their cytosol is thin, their protrusions are branched, and the cell surface expresses CD163, CD206, and arginase-1 (Arg-1) [42]. In contrast to pro-inflammatory microglia, M2-type microglia can release anti-inflammatory cytokines (IL-4, IL-13, IL-10, and TGF-β), growth factors (insulin-like growth factor 1), and neurotrophic mediators (brain-derived neurotrophic factor) to help reduce inflammation and promote neuronal survival [43,44].

### 2.2. The Activation and Polarization of Microglia in DR

The activation of microglia is stimulated by extracellular signals, among which glucose and its derivative metabolites figure prominently [45,46,47,48]. Chronic hyperglycemia activates complex pathophysiologic processes in DR, comprising ischemia, hypoxia, dyslipidemia, and advanced glycation end products (AGEs). It is recognized that sustained exposure to hyperglycemia significantly enhances the non-enzymatic glycosylation of large molecules such as proteins and lipids, ultimately leading to the accumulation of AGEs [49,50]. A comparative investigation of human brain cell populations has elucidated that the toxic AGE–albumin compound is mainly produced by microglia, with the synthesis rate of AGE–albumin being increased upon oxidative stress [51]. Wang et al.’s findings imply that AGEs are elevated in the serum of diabetic rats and may be associated with the activation of retinal microglia [52]. Hyperglycemia during early-stage diabetes induces BRB, leading to an augmentation of glucose and AGE levels due to vascular leakage. The efflux of AGEs may directly promote retinal microglial activation through the AGE receptor (RAGE) present on the microglial membrane, triggering the activation of NOX and thereby augmenting the intracellular synthesis of ROS [53]. In the feedback, ROS enhancement, in turn, may contribute to AGE formation [54] and RAGE expression [55].

In recent years, an abundance of research has shown that environmental perturbations in the retina activate microglia and contribute to retinal inflammation in DR [56,57]. Several animal models have been created to verify the polarization tendency of retinal microglia in DR. In a db/db mouse model, there was an increase in arginase-1/Iba-1 double immunopositive cells in the early stages of DR (5 weeks of age) [58]. This result suggests that the M2 anti-inflammatory phenotype is already present in microglia during the early stages of DR. With the prolongation of the disease course, at 8 weeks of age, the decrease of arginase-1 immunostaining and the high expression of iNOS suggested the possibility of microglia polarizing into the M1 state. More importantly, in addition to animal experiments, clinical studies have also shown that the appearance of retinal microvascular lesions in DR may be related to reduced M2 polarization and an increased M1/M2 ratio [59,60]. Since in vitro M1-polarized human adult microglia express the distinctive markers CD74, Iba-1, and iNOS [61], Ana et al. analyzed these markers in retinas from post-mortem diabetic and non-diabetic donors [58]. They found that the mRNA levels of Iba-1, CD74, and iNOS were markedly elevated in the retinas of diabetic donors, which revealed the transformation of microglia to the M1 type in patients with DR.

All in all, targeting microglia may be a strategy to treat DR, but the effectiveness of targeted therapy is rarely reported. Recent studies have shown that the synthetic progesterone analogue norgestrel reduces the CD68^+^ M1 phenotype in the rd10 model of retinitis pigmentosa through the presence of progesterone receptors on microglia cells and causes the expression of CD206/MRC1, arginase, etc. [62] Thus, it transforms into an M2 phenotype, ultimately preserving the activity and visual function of retinal photoreceptor cells. These observations may suggest that by converting microglia into an M2-polarized state, neuroinflammation in the early stage of DR could be alleviated, thereby reducing retinal blood vessel damage and neuronal degeneration, which may provide new insights for targeted treatment of DR.

### 2.3. The Immunometabolism of Microglia in DR

In recent years, the emerging literature on immunometabolism is defining new roles for cellular metabolic pathways in microglia polarization in DR [25,45,46,48,63]. Immunometabolism indicates the metabolic processes within immune cells, thereby influencing the destiny and functional outcomes of these cellular entities [64,65,66].

Under physiological conditions, the energy required by microglia mainly comes from the decomposition process of nutrients such as sugars, fats, and proteins, among which the tricarboxylic acid (TCA) cycle and oxidative phosphorylation (OXPHOS) of glucose are the most important processes [67]. However, this metabolic process is not constant. In previous studies, tumor cells still preferentially use glycolysis to produce lactic acid for energy under normal oxidation conditions rather than OXPHOS through aerobic respiration, a process known as the Warburg effect [68,69]. Similar to the “aerobic glycolysis” of tumor cells, the chemotaxis, phagocytosis, and proliferation of microglia after being activated into M1 type require dynamic recombination of the actin cytoskeleton and secretion of cytokines, so that M1 type microglia are in a state of energy deficiency. Although glycolysis produces much less ATP (2 ATP) than mitochondrial OXPHOS (36 ATP), glycolysis can be activated more quickly to match the energy demand of M1 microglia. In contrast, M2-type microglia, which are involved in anti-inflammatory repair, require a large amount of stable and sustained energy through mitochondrial OXPHOS to support normal life activities. During this process, the alterations in the expression levels and functionality of glucose transporters on the membranes of microglial cells are instrumental in the elevated glucose concentrations within the retina. Several reports have indicated that glucose uptake in microglia is predominately mediated by glucose transporter (GLUT) 1, especially under inflammatory conditions [70,71,72]. However, when treated with STF31, which impedes GLUT1-mediated glucose uptake in inflamed microglia, the metabolic pathway can be reprogrammed towards OXPHOS [73]. Therefore, targeting GLUT1 may emerge as an effective way to prevent DR.

As we mentioned earlier, integrated multi-omics reveals that activated microglia with an IL-1β increase in the early DR contribute to a metabolic bias favoring glycolysis, purine metabolism, and triacylglycerol synthesis, but less TCA. By establishing hypoxic-stimulated microglia cell line BV2, we observed that cytokine-related gene sets, including IL-1β, were significantly upregulated along with glycolysis gene sets [74]. These findings may indicate that activated microglia with intracellular metabolic reprogramming, especially glycolysis, in the retina, may contribute to pro-inflammation in the early DR. Similarly, in a mouse model of oxygen-induced retinopathy (OIR), high glycolysis of retinal microglia and endothelial cells is involved in the formation of retinal pathological blood vessels. Conversely, the knockout of glycolytic activators 6 phosphofructo 2 kinase/fructose 2,6 bisphosphatase (PFKFB3) in microglia inhibits the ability of microglia to release pro-inflammatory and pro-angiogenic factors and their transformation into pathological retinal angiogenesis-associated glycolytic microglia [75]. Moreover, the increased glycolytic activity of DR microglia appears to be more than a result of the proinflammatory state and may actually be a prerequisite for transformation into M1 proinflammatory microglia. As is demonstrated by two independent laboratories, blocking glycolysis with the glucose analogue 2-deoxyglucose can significantly inhibit pro-inflammatory microglial activation in vitro, which reduces the LPS-mediated increases in IL-6, IL-1β, and nitric oxide synthase 2 [76,77]. Taken together, these findings strongly support that changes in the metabolism of microglial cells in DR are closely related to the maintenance of their polarization state.

## 3. The Redox-Mediated Immunometabolic Regulation of Microglial Activation

**Figure 1:**
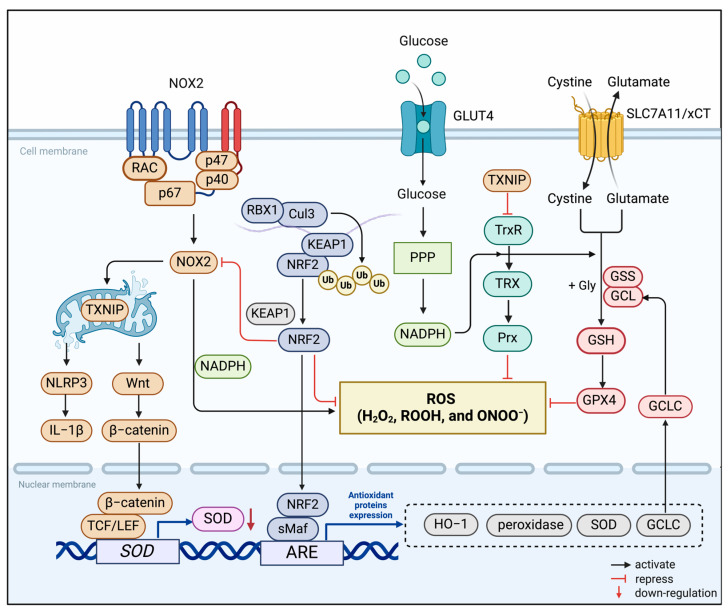
Redox systems involved in microglia. The NADPH Oxidase (NOX), thioredoxin (TRX), and nuclear factor E2-related factor 2 (NRF2) systems constitute the redox homeostasis of the microglia’s internal environment. NOX2 is the main source of ROS generation in microglia. It reduces oxygen molecules to O_2_^−^ or H_2_O_2_ by single-electron reduction dependent on NADPH. TRX-associated proteins are disulfide reductases that regulate the cellular redox state. Induced by various oxidative stresses, the TRX system scavenges ROS in association with TRX-dependent peroxiredoxins. As an endogenous inhibitor of the TRX system, thioredoxin-interacting protein (TXNIP) is transferred to the mitochondria after induction by ROS produced by NOX2. It activates the inflammatory body NOD-like receptor thermal protein domain associated protein 3 (NLRP3) and induces microglia to release the proinflammatory mediator IL-1β. In the meantime, the activation of the Wnt/β-catenin signaling pathway down-regulates the expression of antioxidant enzymes in microglia, further aggravating cellular oxidative stress. The transcription factor NRF2 binds to Kelch-like ECH-associated protein 1 (KEAP1) in the resting state and is fixed in the cytoplasm. Stimulated by oxidative stress, it separates from KEAP1 and translocates into the nucleus, binds to antioxidant response elements (ARE), and regulates the transcription-induced expression of multiple antioxidant genes.

In biological systems, redox reactions occur by transferring electrons from reduced donor molecules to acceptor molecules. During OXPHOS, the electron transport chain (ETC) transports electrons to molecular oxygen, and oxidative phosphorylation drives energy production in the mitochondria of aerobic organisms [78]. This inevitably produces ROS, such as superoxide anion (O_2_^−^) and hydrogen peroxide (H_2_O_2_) [79,80]. Microglia are major sources of free radicals such as superoxide in the retina and play crucial roles in various retinal diseases, especially DR [81,82]. In this process, some redox systems regulate the homeostasis of the redox microenvironment in microglia (Figure 1). Accumulating reports have shown that energy metabolism and redox state in microglia are tightly interwoven and interdependent to regulate the function of microglia ultimately [83], while the specific mechanism remains to be elucidated.

### 3.1. Redox Systems in Microglia

#### 3.1.1. NADPH Oxidase (NOX) System

NOX is the main functional enzyme for ROS production in the microglia [84]. It reduces oxygen molecules to O^2−^ or H_2_O_2_ via NADPH-dependent single electron reduction, meanwhile generating ROS. The NOX family consists of seven isomers, including NOX1, NOX2, NOX3, NOX4, NOX5, Duox1, and Duox2 [85]. Most of them are located in different subcellular compartments within microglia [86,87,88]. Among them, NOX2, originally thought to be a phagocytic enzyme, is the first identified and most frequently explored in the field of DR research [89,90]. It is conventionally composed of membrane-bound catalytic subunit NOX2, regulatory small subunit p22phox, cytosolic subunits p47phox and p67phox, and a small G protein, Rac1 [91]. Numerous studies have shown that ROS generated by NOX2 can lead to microvascular dysfunction in the early stages of DR, possibly through specific mechanisms: (1) Positive feedback between high mobility group box-1 (HMGB1) and NOX-derived ROS mediates the diabetes-induced up-regulation of retinal apoptotic markers (e.g., PARP-1 and caspase-3), which leads to retinal apoptosis, causing vasculopathy and neuropathy [92]. (2) NOX2 leads to premature senescence of retinal endothelial cells through increased ROS formation, elevated arginase expression and activity, and decreased NO formation [93]. (3) NOX2 is highly activated in retinal cells, enhancing the expression of ICAM-1 and VEGF. This caused an increase in vascular permeability as well as leukocyte adhesion to the vessel wall [94]. In addition to NOX2, another isomer, NOX4, is also thought to be involved in diabetic retinopathy. On the one hand, it exacerbates the permeability and inflammatory response of the retinal vasculature [95], and on the other hand, it promotes retinal neovascularization through the H_2_O_2_/VEGFR2/ERK signaling pathway, which also plays a crucial role in proliferative DR [96]. Intriguingly, differing from NOX2 and NOX4, NOX1-derived ROS cause oxidative damage to the mitochondria of retinal endothelial cells in a high-glucose environment and trigger metabolic memory, giving rise to the progression of DR [97]. Thus, targeting the NOX system in microglia to reduce the toxic effects of derived ROS on retinal cells may be a potential way to reduce diabetic retinal damage.

#### 3.1.2. Thioredoxin (TRX) System

The TRX system, consisting of TRX, thioredoxin reductase (TrxR), and peroxiredoxin (Prx), is an essential antioxidant system that regulates the protein dithiol/disulfide balance through the activity of disulfide reductase to resist oxidative stress [98]. TrxRs is a FAD-containing pyridine nucleotide disulfide oxidoreductase that uses NADPH to reduce the disulfide at the active site of TRXs [99]. Mammalian microglia have two TRX systems, with the TRX1 site in the cytoplasm and the TRX2 site in the mitochondria [83]. The function of TRX is to reduce oxidized cysteine (Cys) residues and break the disulfide bond by transferring electrons to Prxs and some redox-sensitive transcription factors. Prxs are widely distributed in different organs, including the retina, which receives electrons delivered by the TRX system to remove H_2_O_2_, ROOH, and ONOO^−^ [100,101]. Meanwhile, as endogenous inhibitors of TRX, the cys63 and cys247 sites of thioredoxin-interacting protein (TXNIP) can form mixed disulfide bonds with thiols at the active site of TRX [102]. Various reports have pointed out that TXNIP plays a crucial role in glucose metabolism. Ao et al. confirmed that TXNIP is upregulated in both diabetic retinopathy and high glucose conditions [103]. In addition, TXNIP plays a number of physiological roles independent of TRX. TXNIP is likely transferred to the mitochondria after induction by ROS produced by NOX2 [104,105]. It activates the inflammatory body NOD-like receptor thermal protein domain associated protein 3 (NLRP3) and induces microglia to release the proinflammatory mediator IL-1β [106,107]. Recently, taxifolin has been reported to inhibit the inflammatory response of high-glucose-stimulated microglia in mice by attenuating the TXNIP–NLRP3 axis [108]. Therefore, we hypothesize that therapies targeting TXNIP may improve symptoms associated with DR by reducing intracellular ROS levels and neuroinflammation.

#### 3.1.3. NRF2/KEAP Signaling

To cope with hyperglycemia, microglia are equipped with nuclear factor E2-related factor 2 (NRF2) in addition to antioxidant defenses in the organism [109]. NRF2 belongs to the basic leucine zip transcription factors and is a key player in the gene regulatory network of redox homeostasis [110,111]. In the resting state, NRF2 binds to Kelch-like ECH-associated protein 1 (KEAP1) and is fixed in the cytoplasm. When the body is oxidatively stressed, it separates from KEAP1 and translocates into the nucleus for transcriptional activity [112]. Modification of cysteine residue 151 (Cys_151_) of KEAP1, the primary sensor for sensing oxidative stress, alters the conformation of KEAP1 and inhibits ubiquitination of NRF2 [113]. Studies have shown that in diabetic retinal diseases, the redox sensing capacity of KEAP1 is altered due to increased oxidative stress, which prevents the separation of NRF2 from the KEAP1–NRF2 complex [109]. After entering the nucleus, stable NRF2 binds to antioxidant response elements (ARE) responsible for regulating the transcription and induced expression of a variety of genes, including antioxidant defense systems and inflammatory factors [114]. There are more than 250 NRF2-targeted genes, including NAD(P)H, heme oxygenase-1 (HO-1), peroxidase, superoxide dismutase (SOD), and glutamate cysteine ligase (GCL) [115]. Intriguingly, GCL is a heterodimer enzyme with catalytic and modified subunits that has been shown to be a key enzyme in catalyzing the synthesis of the antioxidant glutathione (GSH) from glutamate, cysteine, and glycine. The promoters of the GCL subunit contain ARE3 and ARE4, and NRF2 regulates GCL transcription by binding to ARE4. In diabetic retinas, NRF2 binding and transcription levels at the GCL promoter decrease, resulting in reduced GSH production. Therefore, targeting NRF2/KEAP1/GCL/GSH signals seems to be an effective means to correct the down-regulation of GSH levels in DR.

### 3.2. Redox Signaling Regulates Immunometabolism of Microglia in DR

#### 3.2.1. Redox Regulation of Immunometabolism in M1 Microglia

**Figure 2:**
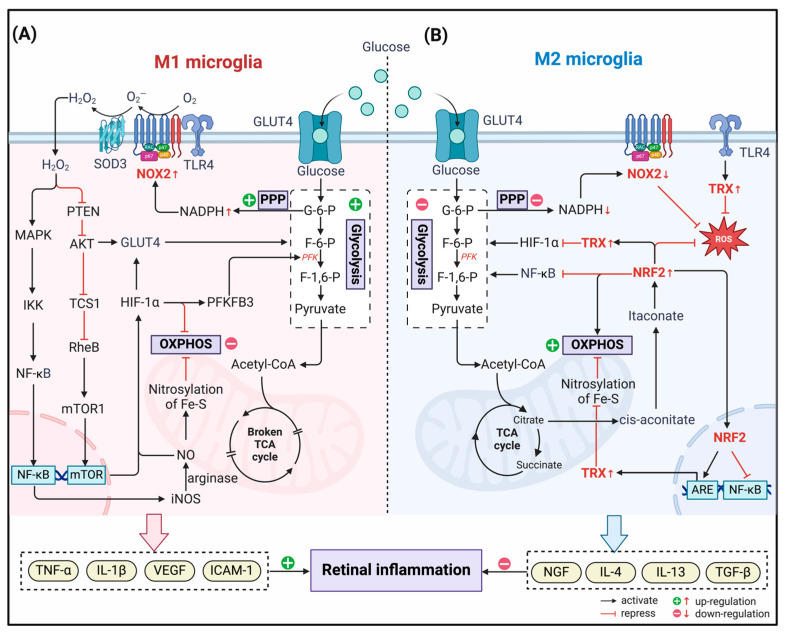
Redox regulation of immunometabolism in the polarization of microglia in DR. (**A**) In M1 microglia, NADPH Oxidase (NOX) 2 couples to toll-like receptor 4 (TRL4) and generates H_2_O_2_ with the assistance of superoxide dismutase (SOD). H_2_O_2_, which enters the cytoplasm, acts as a second messenger. On the one hand, it activates NF-κB through the MAPK pathway, induces up-regulation of inducible nitric oxide synthase (iNOS) expression, and generates NO catalyzed by arginase, which nitrites Fe-S clusters in the mitochondrion and inhibits oxidative phosphorylation (OXPHOS) activity. On the other hand, by inhibiting phosphatase and tensin homolog (PTEN), H_2_O_2_ indirectly activates the AKT pathway, inducing glucose transporter (GLUT) 4 to transfer to the cell membrane and promoting glycolysis. Simultaneously, the mTOR signaling pathway is also activated, which contributes to the elevated expression of hypoxia-inducible factor (HIF)-1α. HIF-1α gained stability by nitroxylation and upregulated the expression of glycolysis-related genes, such as GLUT4 and 6 phosphofructo 2 kinase/fructose 2,6 bisphosphatase (PFKFB3), further promoting glycolysis. (**B**) In M2 microglia, NOX2 activity is reduced due to inhibition of the PPP pathway and decreased NADPH production. At the same time, the accumulated TCA cycle product citrate is isomerized in the cytoplasm to generate itaconate, which can covalently modify Kelch-like ECH-associated protein 1 (KEAP1) and activate nuclear factor E2-related factor 2 (NRF2). NRF2 enters the nucleus and, on the one hand, inhibits the transcriptional activity of NF-κB and reduces the production of pro-inflammatory mediators. On the other hand, it binds to ARE and induces the transcription of antioxidant molecule genes, especially thioredoxin (TRX). The activated TRX system can increase the efficiency of OXPHOS by removing the nitrosylation of Fe-S clusters, while down-regulating HIF-1α-induced glycolytic gene expression to inhibit the process of glycolysis.

During the period of DR, NOX2 is highly active in microglia [89,90]. Notably, polarization of M1 microglia during early inflammation of DR is dependent on metabolic alterations caused by NOX2 activation (Figure 2). It has been demonstrated in traumatic brain injury that NOX2 deficiency significantly reduces pro-inflammatory activation of microglia through an IL-10/STAT3-dependent mechanism, leading to an increase in the anti-inflammatory response [116]. Several inflammatory receptors in microglia are considered to couple with NOX2, including TLR4, which responds to bacterial LPS and pro-inflammatory cytokines [117,118]. After TLR4 is activated, NOX2 coupled with TLR4 is activated, and the superoxide anion radical (O_2_^●−^) is produced at the same time. O_2_^●−^ can mutate into H_2_O_2_ under the mediation of SOD3 on the cell surface, which can freely penetrate the cell membrane to perform the second messenger function [119]. Intracellular H_2_O_2_ is a potent inhibitor of the protein tyrosine phosphatase (PTP), including phosphatase and tensin homolog (PTEN), a redox-sensitive protein [120,121]. Interestingly, PTEN is an inhibitor of AKT/PKB signaling activation [122,123], so AKT signaling increases when PTEN is inactivated, inhibiting microglia polarization from M1 to M2. Specifically, H_2_O_2_ produced by NOX2 inhibits PTEN activity under oxidative stress conditions. The activated AKT pathway promotes glucose absorption by upregulating the expression of GLUT4 on the membrane, thereby promoting microglial glycolysis [124]. In addition, the H_2_O_2_ signal activates the MAPK cascade simultaneously, triggering phosphorylation of IκK, degradation of IκB, and release of active NF-κB [125]. As a result, the secretion of iNOS and proinflammatory mediators increased. In the presence of arginine, iNOS catalyzes the formation of NO. NO produces high levels of peroxynitrite in mitochondria and can promote the nitrosylation of Fe–S clusters in the ETC, inhibit OXPHOS, and promote the occurrence of glycolysis.

In this process, hypoxia-inducible factor-1 α (HIF-1α) appears to coordinate redox signaling with microglial metabolic pathways and is thought to play a key role in DR. Following H_2_O_2_ produced by NOX2, HIF1-α is activated and stabilized under two oxidative regulatory pathways [126]. On the one hand, phosphorylated AKT signaling inactivates the tuberous sclerosis complex (TSC) ½ and mediates the accumulation of Ras homology (Rheb) in the form of GTP-binding, thereby activating the mammalian target of rapamycin complex 1 (mTORC1) and indirectly inducing HIF-1α upregulation [127,128]. On the other hand, due to the accumulation of nitrite in mitochondria caused by the increase of NO, Cys 533 was nitrated in the oxygen-dependent degradation domain of HIF-1α. HIF-1α therefore cannot be degraded by ubiquitination, thus achieving stability [129]. With the increase in HIF-1α transcriptional activity, the expression of glycolytic-related metabolic genes (hexokinase, PFKFB3, GLUT1, etc.) was up-regulated [130]. In addition, HIF-1α can also mediate the up-regulation of pyruvate dehydrogenase kinase 1 and the down-regulation of pyruvate dehydrogenase, thereby inhibiting the conversion of pyruvate to lactic acid and promoting the process of glycolysis [131,132]. In summary, NOX2-guided redox signaling induces microglia to transform in glycolytic and pro-inflammatory directions, which, to a large extent, further aggravates retinal vascular inflammation and nerve damage in DR.

#### 3.2.2. Redox Regulation of Immunometabolism in M2 Microglia

As disease progresses into the middle to late stages, ROS production becomes less important. ATP and NO are depleted in microglia at this point, leading to a progressive accumulation of metabolites such as lactate, NAD+, and AMP. These metabolites send signals of nutrient deficiency and upregulate the expression of antioxidant genes to switch the metabolic phenotype of macrophages. Microglia then shift to the M2 phenotype and possess the ability to resist excessive neuroinflammation and promote tissue remodeling.

We have described that TCA cycling is restricted in M1 microglia, which gives rise to a large accumulation of metabolic substrates, especially citrate. Accumulated citrate is isomerized by aconitase to generate cis-aconitate, which is subsequently decarboxylated by immune-responsive gene 1 to generate itaconate [133,134]. Numerous studies have demonstrated the ability of itaconate to inhibit glycolysis through covalently modifying key glycolytic enzymes [135]. More importantly, itaconate can activate NRF2 by alkylating KEAP1 cysteine residues [136,137]. Activated NRF2 then fights oxidative stress by inducing the production of antioxidant molecules such as GSH, TRX, and SOD. At the same time, the activation of NRF2 is coupled with the inhibition of NF-κB, which inhibits the proinflammatory properties of microglia, including the production of NLRP3 and NO [138]. As another powerful antioxidant system, TRX is also activated and plays an essential role in the removal of nitro and mercaptan. It improves the efficiency of OXPHOS by decreasing the nitrite of Fe–S clusters within mitochondria and reducing the glycolysis flux by inhibiting the expression of the HIF-1α-induced glycolysis gene [139]. Overall, under the modulation of these antioxidant molecules, M2-type microglia exhibit low glycolysis and high OXPHOS and TCA properties for anti-inflammatory efficacy, which is undoubtedly beneficial for the cure of DR.

## 4. Promising Therapies for Diabetic Retinopathy Targeting Redox Regulation of Immunometabolism (Table 1)

### 4.1. NOX System Inhibitors

As previously described, in the DR, the NOX system coupled with TLR causes alterations in microglia immunometabolism through the AKT and MAPK signaling pathways, which, in turn, affect their polarization process. The down-regulation of the NOX system can effectively prevent the occurrence of retinal neovascularization and retinal oxidative stress. Therefore, inhibitors against the NOX system are naturally considered potential drug targets for the treatment of diabetic retinopathy.

GKT137831, also known as setanaxib, is currently recognized as a specific inhibitor of NOX1/4. Since they are structurally similar to NOX, GKT137831 may exert a competitive inhibitory effect on NOX1/4, although the exact mechanism has not been clarified [140]. Several studies have currently demonstrated the potential therapeutic effect of GKT137831 on diabetic retinopathy. Appukuttan et al. demonstrated that GKT137831 treatment significantly reduced dimethyl oxalylglycine (DMOG)-induced ROS generation and VEGFA expression in primary cultured human retinal endothelial cells [141]. Similarly, Jiao et al. also reported that treatment with GKT137831 significantly suppressed ROS levels and apoptosis, as well as caspase-3 activity in HREC, compared to the high-glucose (HG) group [142]. Among other retinal models, Wilkinson-Berka et al. described the beneficial effects of GKT137831 on OIR in rats in two reports [143]. Treatment with GKT137831 significantly reduced Iba1-positive microglia and decreased hypoxia-induced microglia production of ROS, inflammatory molecules (VEGF, IL-6, and TNFα), and leukocyte-recruiting molecules (MCP-1, RANTES, CINC2, CINC3, CXCL3, CXCL5, ICAM-1, and VCAM-1), which is suggestive of the drug’s ability to inhibit the strong pro-inflammatory signaling produced by activated retinal microglia. Currently, GKT137831 has already been administered to human subjects with diabetic nephropathy in a phase II clinical trial [144]. Although the progress of GKT137831 treatment in clinical trials in DR is relatively slow, its therapeutic potential cannot be ignored.

Another novel NOX inhibitor, GLX7013114, has been reported to have improved pharmacological properties in terms of efficacy and specificity in inhibiting NOX4. It has no affinity for the other NOX1, NOX2, and NOX5 isomers present in the retina. This highly selective NOX4 inhibitor has been shown to protect islet cells from cytokines and high glucose [145]. The latest study demonstrated that in STZ-induced rat DR models, topical administration of GLX7013114 prevented early events of DR associated with nitrative oxidative stress and apoptotic cell death [146]. More importantly, the administration of GLX7013114 reversed the activation of diabetic retinal microglia and reduced the expression of pro-inflammatory cytokines in the diabetic retina, thereby protecting the function of retinal neurons and retinal ganglion cells. This suggests the potential of GLX7013114 as a promising therapeutic candidate for the early treatment of DR, although a large number of clinical trials are required to confirm it.

### 4.2. TRX System Agonists

The TRX system is one of the important sulfur-dependent electron donors within the cell, fighting intracellular oxidative stress through disulfide reductase, which is essential for maintaining the oxidation/reduction balance of microglia. However, as an endogenous inhibitor of the TRX system, TXNIP induces oxidative stress by inhibiting the activity of TRX through direct binding to TRX1 and TRX2. It has been previously demonstrated that downregulation of TXNIP in vivo and in vitro reduced ROS production in microglia and alleviated retinal apoptosis [102,147]. These findings suggest that TXNIP may serve as a new potential therapeutic target for diabetic retinopathy.

Verapamil, an L-type calcium channel blocker, reduces the inward flow of extracellular calcium ions, which leads to vasodilation, lowers blood pressure, reduces myocardial contraction, and slows atrioventricular conduction [148,149]. Recent studies have found that Verapamil, the drug of choice for hypertension, has an equally positive impact on the treatment of diabetes and its serious complications, especially DR. Studies have demonstrated that verapamil can reduce intracellular calcium ion levels and reduce carb response element binding protein (ChREBP) into the nucleus, preventing its binding to TXNIP promoter E-box repeats, thereby inhibiting the transcription of TXNIP [150]. Eissa et al. also found that verapamil treatment enhanced TRX-R activity, significantly inhibited TLR4- and TXNIP-mediated NLRP3-inflammasome assembly, and subsequently reduced the release of inflammatory markers (TNF-α and IL-1β) into the vitreous humor and inhibited pathological angiogenesis [107]. It is worth noting that verapamil hydrochloride was approved by the FDA for the treatment of DR in 2016.

### 4.3. NRF2 Pathway Agonists

Accumulating evidence suggests that NRF2 has potential cytoprotective effects on both neurons and blood vessels in the injured retina [151]. In the DR, increased NRF2 activity in the microglial cytoplasm is expected to prevent the release of oxidative products and inflammatory factors. Many studies have shown that several natural compounds can inhibit oxidative stress and inflammation by targeting NRF2, thereby activating cytoprotective genes associated with antioxidant response elements. Thus, NRF2 may provide a new therapeutic target for the treatment of diabetic retinopathy.

Under normal conditions, NRF2 is kept at low levels because it binds to KEAP1 and is inactivated by KEAP1-dependent ubiquitination. Thus, interfering with KEAP1 activity is a rational strategy for agonizing NRF2 to translocate to the nucleus, where it binds to the ARE and mediates the expression of a range of antioxidant protein genes [152]. Interestingly, this is how some of the proven effective NRF2 activators work. Among them, CDDO-Me (RTA 402), one of the triterpenoids, is considered to be the most potent activator of NRF2 [153,154,155]. By interacting with and deubiquitinating specific cysteine residues on KEAP1, this, in turn, allows NRF2 to accumulate and translocate to the nucleus to bind to the ARE to drive transcription of antioxidant genes [156,157]. Notably, CDDO-Me has shown promise in initial clinical trials in patients with T2D and CKD. Ninety percent of T2D and CKD patients showed improvement in their estimated glomerular filtration rate (eGFR) after relatively short-term treatment with CDDO-Me. This suggests the promise of CDDO-Me as a clinical treatment for NRF2 agonists, although its effectiveness in other complications of diabetes, such as DR, remains to be further validated.

Curcumin is a natural polyphenol compound that has antioxidant and anti-inflammatory effects. Many animal experiments have confirmed that, in addition to acting as a direct antioxidant to remove ROS, curcumin can also act as an indirect antioxidant by mediating the activation of the NRF2 pathway [158,159,160]. According to mass spectrometry data, curcumin can modify Cys151 of KEAP1, resulting in conformational changes that inhibit protein interactions between NRF2 and KEAP1 [161]. Several in vivo and in vitro studies have shown its application in the treatment of diabetes and related complications, including DR [162,163]. In preclinical studies in rats, the use of curcumin, insulin, or combination therapy has been critical in regulating blood sugar, reducing oxidative stress, and improving histopathological damage in STZ-induced diabetic rats [163]. In vitro studies of RPE cells cultured under normal and HG conditions, Claudio et al. found that curcumin prevents HG-induced damage in RPE cells by activating Nrf2/HO-1 signaling involved in ERK pathway regulation [164]. However, to date, there are limited data on the pharmacological activity of curcumin in humans, which warrants further exploration.

**Table 1:** The promising therapies targeting redox regulation of immunometabolism for DR.

Classification	Drug	Current Use in Clinical Diseases	Potential Mechanisms for DR Treatment	References
NOX system inhibitor	Setanaxib (GKT137831)	Diabetic retinopathy, renal complication of diabetes, atherosclerosis, liver fibrosis, and idiopathic pulmonary fibrosis	Competitive inhibitor of NOX1/4. On the one hand, it inhibits retinal neovascularization and glial cell inflammation by modulating the VEGF/VEGFR2 pathway. On the other hand, it also reduces cell death and caspase-3 activity in the DR.	[140,141,142,143,144]
	GLX7013114	Diabetic retinopathy and type 2 diabetes	Highly selective inhibitor of NOX4. On the one hand, topical administration of GLX7013114 prevented early events of DR associated with nitrative oxidative stress and apoptotic cell death. On the other hand, GLX7013114 reversed the activation of diabetic retinal microglial and reduced the expression of pro-inflammatory cytokines in DR.	[145,146]
TRX system agonist	Verapamil	Hypertension and diabetes	Verapamil improved oxidative stress damage in DR by inhibiting TXNIP protein expression, reducing its endogenous inhibition of the TRX system, while restoring beta cell health and improving metabolic abnormalities.	[107,148,149,150]
NRF2 pathway agonist	CDDO-Me	Type 2 diabetes and stage 4 chronic kidney disease	CDDO-Me interacts with specific cysteine residues on KEAP1, causing NRF2 to accumulate and translocate to the nucleus, leading to the up-regulation of ARE-responsive genes and promoting gene expression of antioxidant mole-cules.	[153,154,155,156,157]
	Curcumin	Type 2 diabetes and non-alcoholic fatty liver disease	Curcumin can modify Cys151 of KEAP1, resulting in con-formational changes that inhibit protein interactions between NRF2 and KEAP1.	[158,159,160,161,162,163,164]

## 5. Conclusions

Diabetic retinopathy, one of the most common complications of diabetes, has very limited treatment options currently. Well-recognized anti-VEGF therapies are effective in only one-third to one-half of patients with vision-threatening DR. Therefore, it is necessary to clarify the pathogenic mechanisms of DR and develop new, effective treatments. Accumulating studies have demonstrated that the direction of polarization of microglia in the retina largely influences the progress and prognosis of DR. There is no doubt that M1 microglia contribute to neuroinflammation and vascular injury in the early stages of DR. In contrast, disease remission and tissue healing are highly correlated with M2 microglia polarization. Thus, the goal of our current treatment is to convert microglia from M1 to M2 polarization, although whether this would increase VEGF output and exacerbate the lesions remains debatable.

The emerging literature on immunometabolism is defining a new role for cellular metabolic pathways in microglia reprogramming. In general, the M1-like and M2-like phenotypes of microglia have distinct metabolic profiles and may differentiate into distinct subtypes under the stimulus of chronic metabolic disorders. Therefore, regulation targeting microglial metabolic profiles may be a promising approach to moderate microglial cell polarization and function. In this process, redox signaling is gradually gaining attention. Redox response systems (including NOX, TRX, and NRF2) within microglia regulate cellular polarization direction and function by altering the metabolic balance of glycolysis and OXPHOS. Crosstalk between redox regulation and immunometabolism within microglia appears to open new doors for determining the fate of microglia in DR.

Although we have summarized the underlying mechanisms of redox regulation in microglial immunometabolism within the context of DR, numerous aspects remain to be further investigated. For instance, the AKT/mTOR pathway, which is instrumental in regulating the glycolytic activity of microglia via the NOX2 system [165], also triggers autophagy in various cell types, as documented in references [166,167]. Whether mTOR-induced autophagy is involved in the hyperglycolytic metabolism of microglia remains an open question. Furthermore, in the present review, our focus is on the oxidative stress generated within microglia cells themselves. It is noteworthy to consider the external sources of oxidative stress that contribute to damage beyond the microglia cell population during DR. Du et al. showed that the elimination of nerve photoreceptor cells in diabetes suppresses oxidative stress and inflammatory changes that lead to DR vasculopathy, demonstrating these cells also play an important role in the diabetes-induced increase in retinal superoxide and local inflammation in mice [168]. Similarly, in the rd1 mouse model, Daniela et al. emphasized that only photoreceptors undergo apoptosis, instead of the amacrine, bipolar, or horizontal retinal cells [169]. In fact, it has been known for a long time that oxygen is absorbed primarily at the photoreceptor level in cat retinas [170]. Further studies confirmed that the photoreceptors, especially the outer segments (OS), produce massive ROS because of the localization of ectopic enzymes in the respiratory chain [171]. The retinal rod OS disk membrane, despite the lack of mitochondria, performs OXPHOS and expresses ETC and TCA proteins [172,173]. The presence of this extra-mitochondrial oxidative metabolism lays the foundation for OS to be more susceptible to potential oxidative stress damage. However, whether antioxidant therapy targeting oxidative stress damage in the rod OS of the retina is a viable strategy for the early progression of DR remains to be clarified.

To conclude, in this review, we discuss the origin, phenotype, and function of microglia under physiological conditions. The effects of microglia polarization, immunometabolism, and intracellular redox systems on metabolic pathways have also been elaborated. Finally, we summarize potential therapeutic targets for microglial redox regulation, with the aim of providing new insights into emerging therapeutic approaches for DR.

## Data Availability

No new data were created or analyzed in this study. Data sharing is not applicable to this article.
